# Environmental, entomological, socioeconomic and behavioural risk factors for malaria attacks in Amerindian children of Camopi, French Guiana

**DOI:** 10.1186/1475-2875-10-246

**Published:** 2011-08-23

**Authors:** Aurélia Stefani, Matthieu Hanf, Mathieu Nacher, Romain Girod, Bernard Carme

**Affiliations:** 1 EPaT Team (EA3593), UFR de Médecine - Université des Antilles et de la Guyane, Cayenne, French Guiana; 2 Centre d'Investigation Clinique - Epidémiologie Clinique Antilles Guyane (CIE 802 INSERM), Cayenne General Hospital, French Guiana; 3 Unité d'Entomologie Médicale, Institut Pasteur de la Guyane, Cayenne, French Guiana; 4 Laboratoire Hospitalo-Universitaire de Parasitologie Mycologie, Cayenne General Hospital, French Guiana

## Abstract

**Background:**

Malaria is a major health issue in French Guiana. Amerindian communities remain the most affected. A previous study in Camopi highlighted the predominant role of environmental factors in the occurrence of malaria. However, all parameters involved in the transmission were not clearly identified. A new survey was conducted in order to clarify the risk factors for the presence of malaria cases in Camopi.

**Methods:**

An open cohort of children under seven years of age was set up on the basis of biologically confirmed malaria cases for the period 2001-2009. Epidemiological and observational environmental data were collected using two structured questionnaires. Data were analysed with a multiple failures multivariate Cox model. The influence of climate and the river level on malaria incidence was evaluated by time-series analysis. Relationships between *Anopheles darlingi *human biting rates and malaria incidence rates were estimated using Spearman's rank correlation.

**Results:**

The global annual incidence over the nine-year period was 238 per 1,000 for *Plasmodium falciparum*, 514 per 1,000 for *Plasmodium visa *and 21 per 1,000 for mixed infections. The multivariate survival analysis associated higher malaria incidence with living on the Camopi riverside vs. the Oyapock riverside, far from the centre of the Camopi hamlet, in a home with numerous occupants and going to sleep late. On the contrary, living in a house cleared of all vegetation within 50 m and at high distance of the forest were associated with a lower risk. Meteorological and hydrological characteristics appeared to be correlated with malaria incidence with different lags. *Anopheles darlingi *human biting rate was also positively correlated to incident malaria in children one month later.

**Conclusions:**

Malaria incidence in children remains high in young children despite the appearance of immunity in children around three years of age. The closeness environment but also the meteorological parameters play an important role in malaria transmission among children under seven years of age in Camopi.

## Background

Malaria is a major public health problem in French Guiana, a French overseas region located in South America and separated from Brazil and Suriname by the Oyapock and Maroni Rivers, respectively. The Amazon forest covers 94% of the territory. This area is among the most affected by malaria in South America [1]. To date, French Guiana counts around 3,800 malaria cases every year. The overall involvement of *Plasmodium falciparum *and *Plasmodium vivax *appears to be equal, but these two species present an uneven distribution on the territory. Only 2.6% of cases are attributed to *Plasmodium malariae*. Most transmission occurs inland along the rivers, whereas the seashore, where 75% of the inhabitants live, is almost free of transmission [2].

A study in Camopi, an Amerindian village on the Eastern border of French Guiana, reported a mean annual malaria incidence of 486 per 1,000 over the 2000-2002 period and thus labelled the region as a hotspot for malaria [3]. A more recent study using a retrospective cohort design at the same site reported a global incidence of 935 per 1,000 for children under five years of age, 70% of the cases being caused by *P. vivax*. This investigation highlighted the predominant role of environmental factors surrounding households (*i.e. *clearing of vegetation; distances to the river and to the forest). In addition, ethnicity was also identified as an independent risk factor for malaria [4].

*Anopheles darlingi*, a common and efficient vector in the Americas, has been considered as the primary vector in French Guiana for 50 years [5,6]. This vector has the characteristics of being anthropophilic and endo-exophagic. The nycthemeral activity of *An. darlingi *in the coastal area of French Guiana presents a bimodal rhythm with a peak at dusk and one at dawn, superimposed on a nocturnal activity reaching its peak in the middle of the night [7]. This mosquito species is widely distributed in French Guiana and is responsible for malaria transmission in Amerindian villages of the Upper-Maroni on the western border with Suriname [8,9]. In Camopi, longitudinal surveys have been conducted from January 2003 to December 2006. Most of the anopheline species collected were *An. darlingi*, with a mean human biting rate around one bite/person-night that showed marked seasonality with a peak of abundance from April to July [10].

In the present study, the main objective was to determine risk factors for malaria in children under seven, including additional variables from a previous study [4]. Thus biological, ecological, meteorological, hydrological, entomological, socioeconomic and behavioural variables were studied in relation to malaria.

## Methods

### Study area

The study was conducted in Camopi, a village along the Oyapock River which represents the border between France and Brazil. The village consists of a main central hamlet and 28 hamlets within 15 km^2 ^located along the Oyapock and Camopi Rivers. The 1,200 inhabitants are mainly Amerindians of Wayampi and Emerillon ethnic groups, which are respectively located on the Oyapock and Camopi riversides. They live a traditional life and their main occupations are a subsistence slash and burn agriculture [11], hunting, fishing and picking. Crop culture areas are increasing gradually as these Amerindian populations, former nomads, have settled with the creation of structures, such as the health centre and the school. The houses are wood huts, locally called "*carbets"*, which have a roof of palm leaves, steel sheet or tarpaulin. Nevertheless, modern concrete houses are progressively replacing the traditional ones, particularly in the principal hamlet. Camopi is located in the Amazon rainforest. The climate is equatorial with an annual average temperature of 27°C and a high humidity ≥ 80%. In French Guiana, four seasons are identified: the long rainy season from April to June, the long dry season from July to November, the short rainy season from December to February and the short dry season in March. The cumulated precipitation is around 2,700 mm annually. The village is isolated from the inhabited seashore and the journey in dugout canoes to the first main town, Saint-Georges, takes between 4 and 8 hours, depending of the river level and the season. Tourism is not permitted in Camopi and specific authorization is required for all non-residents wishing to reach the village. The health centre, which is located in the main hamlet of Camopi, ensures free early diagnosis and treatment.

### Study design

An open cohort study of children under seven years of age followed from January 1, 2001 to December 31, 2009 was carried out. The exposed population consisted of the totality of the children of Camopi born between January 1, 1994 and December 31, 2008. Malaria data were not censored after the first malaria infection and the children were followed until the day they completed seven years of age. The list of the children was based on the health centre registry which is assumed to be thorough. The children come to the health centre approximately once a month, generally for an illness or for systematic visits (*i.e. *vaccination). Every six months, there is a verification that all newborn children are included in the cohort, that all malaria data is collected and whether children have moved in another hamlet of Camopi or have moved out of Camopi. Data related to children for whom follow-up had been interrupted was right censored at the interruption date. It was assumed that all malaria attacks were recorded at the local health centre due to the isolation of the population and its limited mobility.

### Clinical and parasitological diagnosis

Malaria was defined as fever (temperature ≥ 38°C at the time of consultation or during the previous 48 hours) associated with a positive thin blood smear for *Plasmodium *asexual forms. Blood smears were first examined in Camopi by nurses trained in microscopy and then checked at the parasitological laboratory of Cayenne Hospital. When blood smear examinations were not feasible, rapid diagnostic tests (OptiMAL^® ^test) were performed. The list of all clinical malaria episodes, their date of diagnosis, *Plasmodium *species and parasitaemia (when available) was established.

### Collecting socio-economical and behavioural data

Data were provided by a Knowledge, Attitudes, Practices and Behaviour (KAPB) questionnaire administered to every child's mother. A previous study provided data for children born between January 1, 1996 and December 31, 2005 [4]. In the present study, the cohort was extended to children born between January 1, 1994 and December 31, 2008. Questions about the knowledge of malaria and its transmission, behavioural habits, use of prophylactic measures (use of mosquito nets, domestic insecticides and topical repellents), socioeconomic status (knowledge of French language, education, physical possessions) and slash and burn culture grounds frequentation were asked during the visits.

### Collecting data on the direct household environment

Environmental exposure was evaluated by direct observation of the patients' houses. Every household was geo-localised with a global positioning system (GPS) Magellan^® ^eXplorist™600 and was described (structure of walls and roof). For each *carbet*, distances to the nearest household, to the forest and to the river were provided by GPS or direct measurement for distances < 50 m. The composition of the environment (percentage of water, bare soil, shrubs, slashed vegetation and forest) within the 50 m around them was visually estimated by the interviewer. The presence of a creek in the vicinity and the inundability were assessed. The frequency of intervention of the county mosquito control service (SDD) was also reported on the questionnaire.

### Environmental data: rainfall, temperature and river levels

Rainfall and temperature records were provided by the French meteorological services in French Guiana, which collects data daily from an automatic station in the village of Camopi. Similarly, daily data on the Oyapock River levels were provided by the national department of environmental services and collected by the hydrometric station of Saut Maripa, 90 km downstream from Camopi. Although Saut Maripa lies at a considerable distance from Camopi, river levels at these two locations have been measured simultaneously in the past and these measurements allowed the river levels at Camopi for the period 2001-2009 to be reliably estimated from those recorded at Saut Maripa. The records of daily rainfall, minimum and maximum temperatures, and minimum and maximum river levels were converted into monthly cumulated rainfall, monthly mean of minimum and maximum temperature, and monthly minimum and maximum river levels. Monthly number of days without rain was extracted from daily rainfall records.

### Entomological data

**Figure 1:**
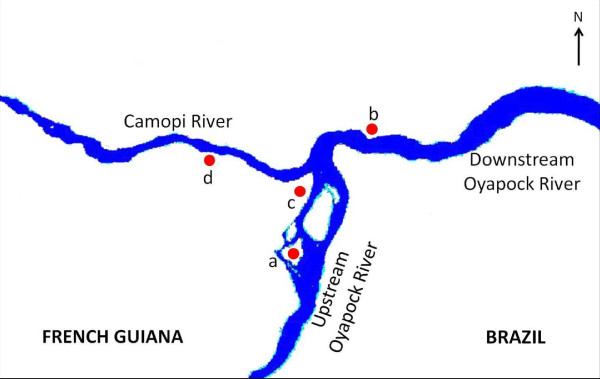
**Groups of mosquito collection sites**. (a) Ilet Moulat (on the upstream Oyapock riverside), (b) Saint Soit (on the downstream Oyapock riverside), (c) Camopi main hamlet (at the confluence of the Oyapock and Camopi Rivers) and (d) hamlets on the Camopi riverside.

Dataset was provided by the Medical Entomology Unit of the Institut Pasteur of French Guiana. Field mosquito collections were made in the main hamlet and in some hamlets along the Camopi and Oyapock Rivers from January 2003 to December 2006. Collecting methodology has been previously described [10]. For the need of the study, collection sites were clustered into four groups represented Figure 1. Human biting rates (HBR), representing the number of female anopheline bites/person-night, were estimated for all four groups of collecting sites.

### Statistical analysis

A survival analysis was used to explore the malaria risk factors. The entry date into the study was January 1, 2001 or the birth date for children born after this date. Exit time was December 31, 2009 or the seventh birthday of the child if he or she reached seven before this date. Time at risk was defined by the entire period from entry to exit. Fifty-seven variables were tested. The influence of each variable on malaria incidence was assessed using the Kaplan-Meier method and Cox modelling. The proportional hazards assumption was tested graphically and with tests based on Schoenfeld residuals. All variables with statistical significance at log-rank test in univariate analysis (p-value < 0.2) were introduced in the multivariate analysis. All models were stratified by year of birth, accounting for age (which violated the proportional hazards assumption) and the period of birth (due to malaria incidence variations according the year). The most parsimonious model was obtained using the log-likelihood ratio test to remove variables from the saturated model. Convergence of this model with both forward and backward automatic stepwise elimination was checked. Survival analysis was made with Stata8^® ^software (StataCorp, College Station, TX).

The associations between malaria incidence and cumulated rainfall, number of days without rain, temperatures and river water levels was investigated by an autoregressive integrated moving average (ARIMA) model. First, a log-transformation was made to stabilise the variance. The optimal combination of ARIMA and seasonal parameters was identified by using the Akaike's information criterion (AIC) [12]. The best-fit model was ARIMA (1,0,0)(0,1,1)_12_. Meteorological factors were tested in univariate analysis, with all factors identified at the 5% level being introduced in the multivariate model. A backwards stepwise approach was used to obtain the final model. Spearman's rank correlation analysis was carried out to assess the association between malaria incidence and meteorological factors identified by the multivariate analysis. In order to examine whether the association between weather and malaria remains constant or whether it is particularly strong in certain months, the response and predictor time-series were also modeled separately and Spearman's rank order correlations between their residuals series at the appropriate lag were examined subsequently. Time-series analysis was realised using the R^® ^software.

Associations between human biting rate and malaria incidence during the same month and the following month were also investigated by calculating Spearman's rank order correlation coefficients for the period 2003-2006, depending on the availability of entomological data. A p-value of < 0.05 was considered indicative of a statistically significant correlation.

### Ethical considerations

The protocol was approved by the CCTIRS (Committee on information processing in research in the field of health) and the CNIL (National commission for computing and liberties). Informed consent was provided by one of the parents of every child before admission into the study. A written consent was signed by the investigator, the respondent parent and the interpreter before the administration of the questionnaire. All malaria cases were treated when diagnosed.

## Results

### Study population characteristics

Five hundred and forty-one children, 288 boys (53%) and 253 girls (47%), were included into the cohort. The ethnic distribution of the children was as follows: 237 Wayampi (44%), 187 Emerillon (34%) and 117 persons of mixed ethnic groups (22%). Regarding spatial distribution, 23% of the children lived in the main hamlet and 37% and 40% of the children lived in the hamlets situated on the Oyapock and Camopi riversides, respectively. The total time at risk was 2279 person-years. During the follow-up, 1,773 malaria cases were observed: 542 caused by *P. falciparum*, 1,171 by *P. vivax*, 48 by mixed infections and 12 by unidentified species. There were 10 deaths during the follow-up but none of them was due to malaria. Children had on average 0.7 malaria episodes per year and the median age for the first malaria attack was 2.3 years. During the study period, 23 children emigrated out of the study area and 25 moved from one hamlet to another. The former were censored at the date of their departure. The latter were censored at the moving date and, at the same date, re-introduced in the study as new individuals, in the new residential hamlet. Thus, the analysis took into account 541 + 25 = 566 individuals.

Most children (75%) spent all their nights under mosquito nets. Around 80% of the interviewed mothers considered malaria as a severe and urgent disease and 57% of them said they consulted the shaman in addition to the health centre's staff. Many families used domestic insecticides and topical repellents (66% and 63%, respectively). Forty seven percent of the mothers mentioned mosquitoes as malaria vectors. Only 32% could name at least one anti-malarial treatment whereas 93% knew at least one malarial symptom (fever was mentioned in 77% of the responses). Considering socio-economic characteristics, 68% of the families owned a dugout canoe and 53% owned an outboard motor. Over 85% of homes had electricity (generator or solar panels) and more than 60% had a television set.

### Inter-annual and intra-annual variations in malaria incidence

**Figure 2:**
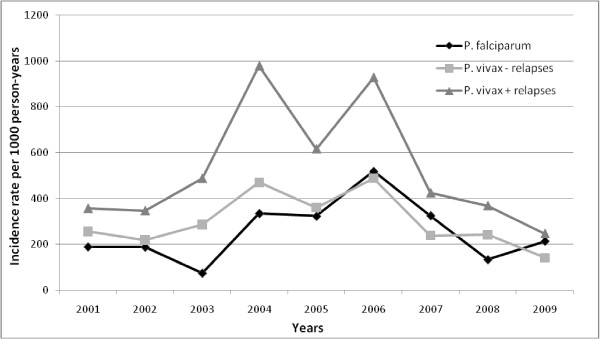
**Inter-annual variations of malaria incidence rates per 1000 person-years attributed to *P. falciparum *and *P. vivax *(including and excluding relapses)**. Incidence rates were calculated for the population 0 - 7 years old.

The overall annual incidence during the study period was 773 per 1,000 person-years. Regarding *Plasmodium *species, the incidences were 238, 514 and 21 per 1,000 person-years for *P. falciparum*, *P. vivax *and mixed infections, respectively. *Plasmodium vivax *relapses in French Guiana have a pure tropical pattern with a short latent period (Chesson strain [13]) [14]. Therefore, for a given child, each *P. vivax *episode occurring within 90 days after another *P. vivax *episode was considered as a relapse. By removing relapses from the database, we focussed on malaria transmission and not on the overall incidence, eliminating the noise. Thus, if relapses due to *P. vivax*, which represent 43% of *P. vivax *attacks, are excluded, the annual transmission incidence rates became 248, 292 and 11 per 1,000 person-years for *P. falciparum*, *P. vivax *and mixed infections, respectively. This varied greatly from year to year, with two peaks of incidence in 2004 and 2006 (Figure 2).

**Figure 3:**
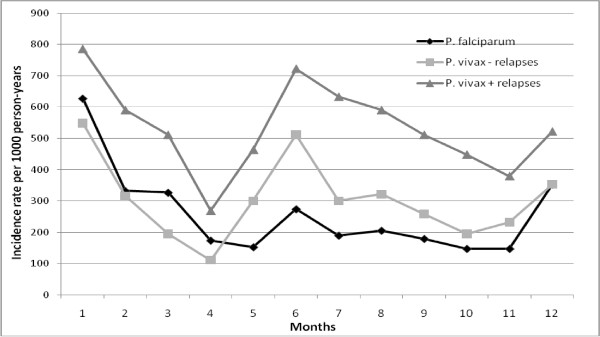
**Intra-annual variations of incidence rates for *P. falciparum *and *P. vivax *(including and excluding relapses) during the period study (2001-2009) in children under 7 years of age**.

When studying the intra-annual variability of the incidence rate, two peaks were observed for *P. vivax*: one in January and another in June, whereas only one high peak was observed in January for *P. falciparum*. (Figure 3).

### Malaria incidence by age

**Figure 4:**
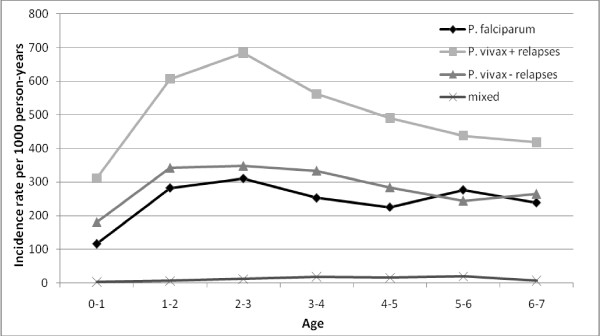
**Incidence rates for *P. falciparum*, *P. vivax *(including and excluding relapses) and mixed infections by age on the study period (2001-2009)**.

From birth to one year of age, the incidence was relatively low (117 per 1,000 and 180 per 1,000 person-years for *P. falciparum *and *P. vivax*, respectively) (Figure 4). The incidence strongly increased up to 2-3 years of age, where the incidence remained the highest (310 per 1,000 and 348 per 1,000 person-years for *P. falciparum *and *P. vivax*, respectively). After this age, the malaria incidence began to decrease until five years of age and remained more or less stable until seven years of age, around 250 per 1,000 person-years for both species.

### Survival analysis

All malaria attacks were analysed regardless of the *Plasmodium *species involved. Among the 57 variables tested, 30 were significantly associated with malaria incidence in university analysis. The multivariate analysis identified six variables associated with occurrence of malaria transmission (Table 1). With regard to the living place, living on the downstream Oyapock riversides or on the Camopi riversides *vs*. on the upstream Oyapock riversides, as living in an isolated hamlet (more than 2 km from the main hamlet) and in a house with more than seven occupants, were associated with higher risk for malaria transmission. Regarding the environmental characteristics of the peridomiciliary space, living in houses cleared of surrounding vegetation within 50 m and living far from the forest were associated with a lower risk of malaria. With regard to behavioural data, going to bed after 7:00 pm. was associated with an increased risk of malaria transmission. Any significant difference of malaria incidence between girls and boys was observed.

**Table 1:** Risk factors for malaria attacks.

Variable	Categories	No. of children	Incidence rate	Hazard ratio	95% CI	p value
River	Upstream Oyapock	262	0.407	1.00		
	Downstream Oyapock	76	0.551	1.35	1.08-1.69	0.008**
	Camopi	228	0.729	1.37	1.13-1.66	0.002**
Percentage cleared around home within 50 metres	<50	150	0.686	1.00		
	50-75	279	0.542	0.81	0.71-0.93	0.002**
	>75	96	0.432	0.84	0.67-1.05	0.119
Distance to Camopi, metres	≤500	137	0.407	1.00		
	501-2000	208	0.451	1.13	0.93-1.38	0.208
	>2000	221	0.754	1.61	1.33-1.94	<0.001**
Distance to the forest, metres	≤50	169	0.737	1.00		
	51-150	202	0.538	0.79	0.68-0.91	0.001**
	>150	156	0.393	0.73	0.60-0.88	0.001**
Number of occupants	<7	223	0.512	1.00		
	7-10	195	0.594	1.17	1.01-1.35	0.037*
	≥11	107	0.591	1.26	1.07-1.48	0.005**
Bedtime, hours	<7:00 pm	103	0.489	1.00		
	≥7:00 pm	354	0.584	1.18	1.01-1.38	0.034*

Multivariate analysis of the risk of malaria transmission using a multiple failure Cox model, stratified for year of birth. The saturated model included 32 variables, which were significant at p < 0.20 in univariate analysis. Analysis time = 2279 person-years, CI = confidence interval. Symbols * and ** correspond to statistical significances at 0.05 and 0.01 first-order risks, respectively.

### Intra-annual variations of climate parameters

**Figure 5:**
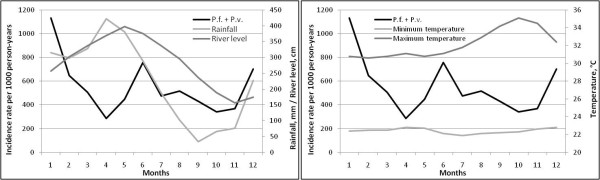
**Intra-annual malaria incidence rates in children and (a) monthly cumulated rainfall and river level, and (b) minimum and maximum temperature in Camopi on the study period (2001-2009)**. Incidence rates have been calculated for the two species combined and *P. vivax *relapses were excluded.

In Camopi, the seasonality is determined by the rainfall, leading to a rainy season from December to June and a dry season from July to November (Figure 5). There is a peak of malaria incidence at the beginning of the rainy season and another one at the end of it. The highest temperatures coincide with the driest months of the year. The lowest temperature (16.4°C) and the lowest mean minimum temperature (20.9°C) were recorded in June 2007. The highest temperature (38,0°C) and the highest mean maximum temperature (36.1°C) were recorded in November 2008. Malaria incidence was significantly higher during the rainy period than during the dry period (p-value < 0.001).

### Association between climatic and hydrologic data and malaria incidence

Time-series analysis revealed nine relevant factors: minimum temperature at time t-3, t-9 and t-11 months; mean minimum temperature at t-1 and t-12 months; mean temperature at t-2 months, the number of days without rain at t-9 months and the maximum river level at t and t-1 month. The multivariate ARIMA model obtained with the inclusion of these nine variables, applying a stepwise descending procedure, is summarized in Table 2.

**Table 2:** ARIMA regression of the monthly malaria incidence in children (2001-2009) against meteorological and hydrological factors in Camopi, French Guiana.

	Coefficient	Standard Error	P value
AR*	0.293	0.110	0.004
SMA**	-1.000	0.275	<0.001
Maximum river level (lag 0)	0.003	0.002	0.020
Maximum river level (lag 1)	0.004	0.002	0.018
Mean of minimum temperature (lag 1)	0.278	0.140	0.023
Mean temperature (lag 2)	0.498	0.171	0.002
Minimum temperature (lag 3)	0.128	0.076	0.046
Minimum temperature (lag 9)	0.160	0.076	0.018
Number of days without rain (lag 9)	0.035	0.015	0.011
Minimum temperature (lag 11)	0.144	0.086	0.046
Mean of minimum temperature (lag 12)	0.355	0.153	0.010

*AR: autoregressive parameter;** SMA: seasonal moving average parameter; AIC of the model = 232.58. Lag scale = months.

**Figure 6:**
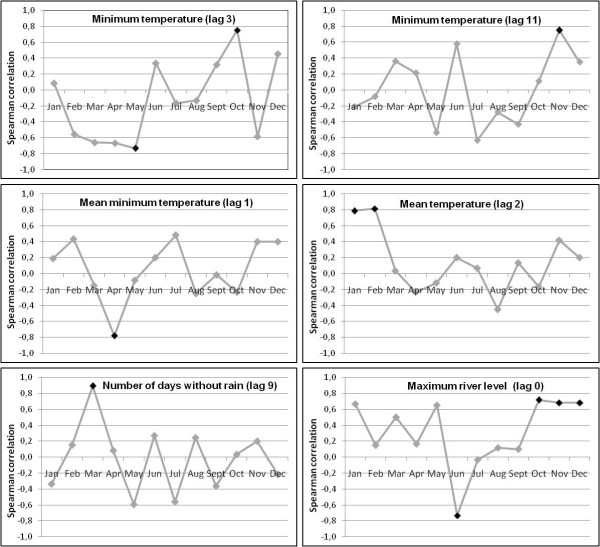
**Spearman's rank order correlation coefficients between residual series of malaria incidence and meteorological factors**. Correlations for which associated P-value < 0.05 are indicated by black markers.

Spearman's correlations between malaria incidence and meteorological factors for which there is at least one statistically significant correlation between residual series are presented in Figure 6. The relationship between malaria incidence and the maximum river level at lag 0 was strongest in June (negative) and in October, November and December (positive). The correlation between incidence and mean minimum temperature one month before was strongest in April. The correlation between incidence and mean temperature two months before was strongest in January and February. The association between incidence and minimum temperature three months earlier was strongest in May (negative) and October (positive) whereas the relationship between incidence and minimum temperature eleven month earlier was strongest in November. The correlation between incidence and number of days without rain nine months before was highest in March.

### Relationship between entomological data and malaria incidence

**Figure 7:**
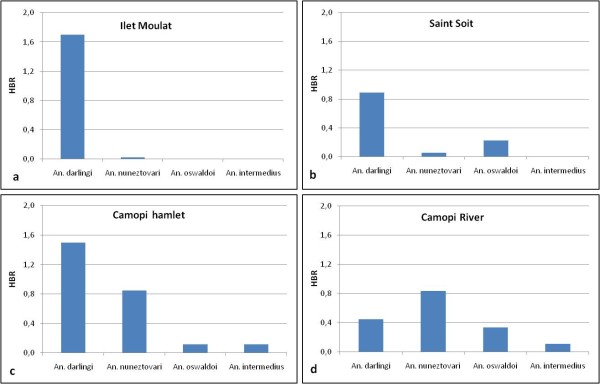
**Human biting rates of anopheline species collected between 2003 and 2006 in different groups of hamlets of the study site**. (a) Ilet Moulat (on the upstream Oyapock riverside), (b) Saint Soit (on the downstream Oyapock riververside), (c) Camopi main hamlet (at the confluence of the Oyapock and Camopi Rivers) and (d) hamlets on the Camopi riverside.

Human biting rates of the different collected anopheline species according to the four groups of hamlets are presented Figure 7. Although *An. darlingi *was the main overall collected species, the *Anopheles nuneztovari *HBR was higher in the hamlets located on the Camopi River. A peak of HBR was observed in May for *An. darlingi *and a lower peak was observed in April-May for *An. nuneztovari*. No other peak of HBR was observed during the year for theses two species. Regarding *An. darlingi *characteristics, a significant correlation was observed between malaria incidence and human biting rate (HBR) recorded one month earlier (p value = 0.03).

## Discussion

During the nine years of the study, the global incidence rate in children of Camopi remained very high with two epidemics in 2004 and 2006, reaching 800 and 1,000 per 1,000 person-years respectively. During the first year of life, the incidence was quite low presumably because the presence of maternal antibodies provides partial immunity. At one year of age, the incidence began to increase sharply to reach a maximum between two and three years old (almost 1,000 per 1,000 person-years). Immunity seems to begin to develop at this age, for both species, but appeared to be mainly protective against *P.vivax *relapses. Amerindian children have different activities as they grow and gain independence in their movements in the village. Moreover, around seven or eight years of age, boys begin to go to the forest for hunting and to the river for fishing whereas girls accompany their mother to slash. Hence, around five years of age, a different exposure may be responsible for the small increase of *P. falciparum*.

The survival analysis showed a strong association between environmental exposure and malaria transmission in children under seven years old. Indeed, the analysis permitted to identify several risk factors linked to the environmental characteristics of the surroundings of the house. Malaria transmission increased with distance from the central main hamlet of Camopi. Therefore, children living in isolated hamlets had an increased risk of malaria. However, a higher number of people in the same home (more than seven occupants) were independently associated with a higher risk of malaria. Human aggregation is likely to increase the probability for vectors near homes to be infected [4,15].

Regarding other environmental factors, the proportion of cleared vegetation within 50 m around the houses was a protective factor for malaria, as previously described. Indeed, this characteristic of land cover is not favourable for the rest of adults and the maintenance of breeding sites. Proximity to the forest was associated with a higher risk of malaria. According to other authors, when houses are located not far from the forest, *An. darlingi *returns to the forest after feeding [16-19]. However, non-environmental factors may be partly responsible for the relation between increasing incidence and the distance from the main hamlet of Camopi. There was a significantly different risk of malaria according to the ethnic group in univariate analysis. A previous study in Camopi found a strong association of ethnicity with first malaria attack, even after adjusting for behavioural and environmental factors [4]. These results could suggest that Emerillon children have higher genetic susceptibility than Wayampi children. However, this phenomenon was not visible in our multivariate analysis, which did not take into account *P. vivax *relapses and thus was then closer to the transmission phenomenon.

Furthermore, another variable is likely to better explain the malaria incidence while being correlated to ethnicity. This is the case of the variable "river" divided in three groups: the upstream Oyapock riverside where the Wayampi live, the downstream Oyapock where the mixed ethnic groups live and the Camopi riverside where the Emerillon live. The behaviour of the residents and the protection measures used also play a role in the incidence of the disease. Indeed, the use of topical repellents and domestic insecticides and interventions of the county mosquito control service were significantly associated with a lower risk of transmission in univariate analysis. In addition, children who used to go to sleep after 7:00 pm had a higher risk of transmission. It is likely that children going to sleep earlier are protected by mosquito net at dusk, when *An. darlingi *reaches its first peak of activity [7].

Climatic and hydrologic variations appeared to have an impact on malaria incidence at relatively short-term (lag 0 to lag 3) and at longer term (lag 9 to lag 12). Considering the short- term effect, some plausible explanations can be put forward. The mean minimum temperature was globally positively associated with malaria incidence one month later and the minimum temperature was positively associated with malaria incidence three months later. Moreover, the mean temperature was positively associated with a higher incidence that occurred two months later, and especially at the beginning of the rainy season. These observations could be explained by the fact that an increase temperature shortens the interval between egg-laying episodes and enhances the larval development. Moreover, a higher temperature is also likely to accelerate the sporogonic cycle of the *Plasmodium*. Thus, a high temperature may allow better survival of vector populations and therefore a higher transmission that could be responsible for an increasing incidence in the following months. Conversely, lower minimum temperatures may be responsible for a decreased incidence, slowing the sporogonic cycle of the parasite and decreasing vector survival. Regarding hydrological factors, incidence rates were positively associated with the maximum river level at the same month and one month earlier. Thus, particularly high water may create flooding on the river bed, leading to the creation of suitable larval breeding sites, particularly at the end of the year. This phenomenon has been observed along the Maroni River and in other countries of Central and South America [20-24]. Thus, an increase in the larval anopheline abundance may increase the malaria transmission related to the adult stage. This has been previously observed at a weekly temporal resolution in Camopi where vector abundance was positively correlated with the river level a few weeks earlier [10].

Regarding the long-term impact of climate on malaria incidence, it is difficult to grasp the meaning of these correlations and the statistical results have to be considered with caution. The significant results could be due to unidentified confounding factors or residual effects of seasonal factors that are not taken into account at a short term. This long-term hypothetical effect has been previously observed in Cacao, French Guiana, where meteorological conditions in a given year may affect malaria in the following year [25]. Nevertheless, the biological impact of meteorological factors on vector populations over a long period can only have hypothetical explanations. Overall, annual climate seasonality was linked to malaria seasonality as observed by others [26,27]. In French Guiana, a global and durable climate anomaly such as El Niño episode is likely to increase malaria [28].

*Anopheles darlingi *human biting rate was correlated to the malaria incidence rate in children one month later. This is consistent with what was previously found in correlating entomological data with malaria incidence in the general population [10]. Nevertheless, none of the 148 specimens of this species collected from January 2003 to December 2006 in Camopi was found naturally infected with *Plasmodium *[10]. This study focused on young children with the hypothesis of a nightly transmission due to the characteristics of *An. darlingi *[5]. Other authors mentioned that *An. darlingi *has a 24 hours activity and can be found outside during the day in French Guiana [7]. Other anopheline species could play a role in the transmission of malaria including during the morning [6,29,30] or in a sylvatic environment around the hamlets [10]. Indeed, *An. nuneztovari *could play a role in the transmission, along the Camopi River where the incidence remains higher. Indeed, this species could be a secondary vector when present in sufficient numbers. This species, exophilic and aggressive on humans, may be collected on humans in large numbers in some Amazonian areas [20,31,32].

Given that it can be assumed that *An. darlingi *transmitted *P. vivax *and *P. falciparum*, it was intriguing to observe that *P. vivax *had a high transmission in May and June whereas *P. falciparum *had a much lower transmission during the last period. A tentative explanation is that the age composition of the *An. darlingi *population may depend on the seasons and the environment [9,33]. Thus, long-lived females that would be good vectors for the two plasmodial species might be responsible for malaria transmission in December and January. On the other hand, females with a lower life expectancy would be poor vectors for *P. falciparum *which has a longer extrinsic cycle than *P. vivax*, and should be responsible for the transmission of *P. vivax *in May and June only. Unfortunately, the comparison of survival rates of females in these two periods could not be performed due to the low numbers of mosquitoes collected. Another explanation is that *P. vivax *relapses in the months following the first peak provide gametocytes to the emerging vector population thus amplifying the *vivax *transmission.

Even if the distribution of malaria is determined by climatic and other geographic factors which affect mosquito and *Plasmodium *reproduction at a given time, malaria is also influenced by environmental changes [34]. Therefore, the impact of deforestation on malaria transmission that has been previously described [18,34-36] should be investigated in this area where the settlement of Amerindian populations coupled with gold-mining activities constantly cause forest openings.

## Conclusions

In Camopi, malaria incidence in children remained very high over the 2001 - 2009 period despite the appearance of immunity around three years of age. *Plasmodium falciparum *and *P. vivax *incidences presented two different intra-annual patterns. The occurrence of malaria is mainly due to the environmental features around the homes, and not so much to individual behaviours or population habits. Although malaria incidence is associated with a complex array of variables, the present data gives a clear picture of where households should not be build, and how the peridomestic space should be kept to minimize the risk of malaria. Moreover, meteorological and hydrological variations such as minimum temperatures and maximum river water level seem to have a relevant impact on malaria incidence in the short term (from zero to three months-lags). Nevertheless, the results of the present study should be carefully extrapolated to the general population because transmission in adolescents and adults is partly different due to a different exposure and a stronger immunity.

## Competing interests

The authors declare that they have no competing interests.

## Authors' contributions

AS participated in the research design, data collection, analysis and interpretation, and prepared the manuscript. MH participated in the data analysis and interpretation, and revised the manuscript. MN provided guidance in data analysis and contributed to data interpretation and manuscript revision. RG was involved in the entomological data interpretation and manuscript revision. BC was responsible for overall scientific management, designed the cohort and was involved in the interpretation of data and manuscript revision. All authors read and approved the final manuscript.
